# Preparation of Eugenol–Thymol–Cuminal Composite Essential Oil Microcapsules with AITC & β-Cyclodextrin Inclusion Complex and Its Effect on Quality of Chilled Pork

**DOI:** 10.3390/foods14061029

**Published:** 2025-03-18

**Authors:** Wenxiao Li, Fan Yang, Li Chen, Ke Ding, Xiangning Chen

**Affiliations:** 1 Department of Food Science and Engineering, Beijing University of Agriculture, Beijing 102206, China; 202330621111@bua.edu.cn (W.L.); sunshine11102@163.com (F.Y.); chenli@bua.edu.cn (L.C.); 19967202@bua.edu.cn (X.C.); 2 Key Laboratory of Agricultural Product Processing and Quality Control (Co-Construction by Ministry and Province), Ministry of Agriculture and Rural Affairs, Beijing 102206, China

**Keywords:** composite plant essential oil microcapsule, allyl isothiocyanate microcapsule, allyl isothiocyanate with β-cyclodextrin inclusion, chilled pork

## Abstract

The preservation of chilled fresh pork is an issue that has widely drawn significant attention. A novel microcapsule was developed in this study, specifically a composite plant essential oil microcapsule (CEO mps) prepared using gum arabic (GA) and an inclusion compound of allyl isothiocyanate (AITC) with β-cyclodextrin (β-CD), in which AITC is encapsulated within the cavity of β-CD molecules. In this formulation, AITC functions as an antibacterial agent, while the essential oils provide antioxidant properties that further enhance bacterial inhibition. The encapsulation ratio of AITC to β-CD was optimized at 1:1, with nuclear magnetic resonance (NMR) hydrogen spectroscopy confirming that AITC was incorporated into β-CD through its wider cavity. The morphology and structure of CEO mps were characterized using Fourier transform infrared spectroscopy (FTIR), scanning electron microscopy (SEM), and laser particle size analysis, and these were compared to those of AITC mps—microcapsules prepared with GA and β-CD as wall materials and AITC as the core material. The results indicated that CEO mps exhibited superior appearance and physical stability in comparison to AITC mps. The release rate of CEO mps was evaluated using gas chromatography–mass spectrometry (GC/MS), revealing sustained release characteristics. On day 12, cumulative releases for AITC, eugenol, cuminal, and thymol were 61.82%, 57.96%, 44.34%, and 38.65%. Finally, the efficacy of CEO mps in preserving chilled pork was assessed by measuring pH levels, total volatile base nitrogen (TVB-N), color parameters (L*, a*, b*), thiobarbituric acid-reactive substances (TBARSs), water loss, and total microbial counts. The results demonstrated that CEO mps significantly inhibited microbial growth in chilled pork, reduced TBARS and TVB-N values, and helped preserve meat color integrity, thereby effectively extending shelf life by approximately six days. Overall, the experimental findings confirmed that the developed CEO mps possess both antibacterial and antioxidant properties, thereby improving both the shelf life and organoleptic quality of chilled pork.

## 1. Introduction

Pork serves as a valuable source of protein, fats, carbohydrates, and essential trace elements for human nutrition [1]. However, improper storage practices can lead to microbial contamination and oxidative rancidity of lipids in pork products [2]. Traditional preservation methods, such as curing, air-drying, or modern freezing techniques, not only influence the flavor profile but also impact the safety of pork consumption [3]. Consequently, there is an urgent need to develop advanced preservation technologies to address these critical issues. The microencapsulation of plant EOs is thus gaining increasing attention as an environmentally friendly method to improve the quality of meat products.

Essential oils (EOs) are natural preservatives that do not have negative environmental impacts. They possess both antibacterial and antioxidant properties. Allyl isothiocyanate (AITC), an active compound found in cruciferous vegetables such as oilseed rape and kale, is a broad-spectrum bacteriostatic agent that inhibits the growth of bacteria and fungi [4], including *Salmonella Typhimurium*, *Listeria monocytogenes Scott A*, *Escherichia coli O157:H7*, *Pseudomonas aeruginosa*, *P. fragi*, *Proteus vulgaris*, *Staphylococcus aureus*, and *Micrococcus luteus* [5]. In 1985, mustard oil was marketed in Japan as a preservative for soy sauce. This suggests that as early as 1985, mustard oil was already being used as a preservative in food products. This fact not only strongly attests to its safety but also provides a solid foundation for the feasibility of the present study. Eugenol has demonstrated a strong inhibitory effect against bacteria such as *Lactobacillus*, *Streptococcus lactis*, *Salmonella Typhimurium*, *Staphylococcus* and fungi such as *Candida albicans*, as well as exhibiting antioxidant activity [5,6]. Thymol has bacteriostatic properties against both Gram-positive (*Staphylococcus aureus*) and Gram-negative (*Escherichia coli* and *Pseudomonas aeruginosa*) bacteria [7], and it can serve as a natural alternative to synthetic fungicides [8]. Additionally, a study showed that thymol significantly inhibited the formation of both primary (peroxide value) and secondary (TBA and thymol glycoside) oxidation products in mayonnaise, making it a promising natural antioxidant [9]. Cuminal inhibits the growth of *Escherichia coli* and *Listeria monocytogenes* [10], with higher concentrations of cuminal exhibiting stronger antioxidant effects [11]. The antioxidant activity of plant EOs is primarily attributed to the presence of phenolic acids (such as gallic acid and protocatechuic acid), phenolic diterpenes (such as hypoglic acid and dehydroagastol), flavonoids (such as quercetin and kaempferol), and volatile oils (such as eugenol, thymol, etc.) as their active components.

Most EOs are derived from aromatic plants, and they are characterized by their distinctive odors and high volatility. As such, their encapsulation alters their physicochemical properties, such as solubility and volatility, which in turn controls their release rate and extends the duration of their activity. Spray drying is a well-established technique for microencapsulation, widely used in the food industry due to its low cost and effective encapsulation performance. The selection of wall materials is crucial in the preparation of microcapsules (mps) by spray drying. Common wall materials such as gum arabic (GA), maltodextrin, and cyclodextrin are frequently used either alone or in combination to enhance the encapsulation efficiency [12,13]. Cyclodextrins were first discovered by Villers in 1891, and their structure was elucidated by Frendenberg and French in 1935 [14]. Cyclodextrin molecules have a hydrophilic outer ring, a hydrophobic inner ring, and a 3D cavity. They can encapsulate various compounds to form inclusion complexes [15]. Among the three types of cyclodextrins α-, β-, and γ-cyclodextrins, β-cyclodextrin (β-CD) has the highest degree of crystallinity.

To achieve the combined benefits of bacteriostatic, antioxidant, and sensory enhancement, the use of composite plant essential oil microcapsule (CEO mps) is considered a viable solution. Traditionally, CEO mps are prepared by mixing various active ingredients as core materials and encapsulating them. However, due to the limited space within the microcapsule, incorporating multiple types of EOs poses a challenge. This limitation reduces the quantity of each EO that can be encapsulated, potentially affecting its efficacy. To address this issue, a novel microencapsulated structure was designed in this study, using gum arabic and β-CD as wall materials. The uniqueness of these wall materials lies in the embedding of AITC molecules within the cavity of each β-CD molecule, forming inclusion complexes. In this configuration, AITC primarily exerts bacterial inhibitory effects, while a composite of three plant EOs—eugenol, thymol, and cuminal—serves as the core material, providing antioxidant and additional antibacterial effects. Upon application, AITC is released from the outer wall of the mps, while the CEO is gradually released from the core, ensuring optimal antioxidant and bacteriostatic effects. It is noteworthy that the use of an AITC&β-CD inclusion complex as a wall material in mps has not been previously reported. Our experimental results demonstrate that mps with this structure exhibit significant preservation effects and show promising potential for practical applications.

## 2. Materials and Methods

### 2.1. Materials

Qualified chilled fresh pork [the part of the animal used was pork tenderloin (M. psoas major), free of bone, visible connective tissue, and external fat] was purchased from Dahongmen Meat Tiantongyuan Store, Changping District, Beijing, China and stored under refrigeration at 4 ± 1 °C. Eugenol (99.9%), cuminal (99.8%), thymol (99.8%), gum arabic (GA) powder, and β-cyclodextrin (β-CD, ≥99.0%) were obtained from Maclean’s (Shanghai, China). Allyl isothiocyanate (AITC, ≥95%) and 2-thiobarbituric acid (98.0%) were purchased from Sigma-Aldrich (St. Louis, MO, USA), while trichloroacetic acid (TCA) was sourced from Tianjin Guangfu Fine Chemical Research Institute (Tianjin, China). Anhydrous ethanol, n-hexane, and other organic solvents were of analytical grade and purchased from Beijing Chemical Industry Co., Ltd. (Beijing, China).

### 2.2. Preparation of AITC & β-CD Inclusion Complexes

An electronic balance was used to accurately weigh 4.00 g of β-CD into a beaker, to which 100 mL of deionized water was added. The beaker was then placed in a constant-temperature magnetic stirrer, and the solution was stirred under a water bath at 60 °C and 500 rpm. Once all the β-CD had dissolved, the solution was removed and cooled to 45 °C, where it remained clear and transparent. Concurrently, 4 mL of allyl isothiocyanate (AITC) and 4 mL of anhydrous ethanol were added to the same beaker using a pipette, ensuring thorough dissolution of the AITC in ethanol. The resulting AITC–ethanol solution was slowly and uniformly added to the β-CD solution using a burette, while stirring continuously at 900 rpm for 4 h. During this process, the solution gradually became turbid, indicating the formation of the β-CD-AITC inclusion complex. The solution was then filtered, and the filter cake was washed alternately with anhydrous ethanol and deionized water at 45 °C to remove any unassociated AITC and β-CD. The resulting white AITC & β-CD inclusion solid was then dried under vacuum at 60 °C for 12 h to obtain the dried AITC & β-CD inclusion powder, which was subsequently used as the wall material for the CEO mps. After measurement, in AITC&β-CD, the content of AITC was 72.97 μL/g. In order to obtain a larger amount of AITC and β-CD at one time, the amounts of the required experimental materials can be proportionally increased according to the needs during the experimental process.

### 2.3. Preparation of CEO Mps

A total of 50.00 g of AITC & β-CD inclusion powder was accurately weighed using an electronic balance, along with an equal mass of gum arabic (GA) powder, which were both placed into the same beaker to serve as the wall material. The total required mass of core material was calculated to be 10.00 g, based on the mass ratio of core material to wall material (1:10). According to the total content of the wall material and the core material (23%), and the moisture content (77%), the required amount of deionized water was calculated to be 368.30 g. The measured deionized water was added to the beaker containing the wall material powder. Next, 2.40 g of Tween-80, at a 0.5% mass ratio, was added to the mixture, and the solution was stirred well with a magnetic stirrer at 550 rpm at room temperature to prepare the wall material solution. To make up for the limitations of AITC in terms of its antioxidant capacity, we introduced three essential oils. Through the comparative experiment on the antioxidant effects of single plant essential oils and compound plant essential oils, as well as the proportion optimization experiment, we finally determined the mass ratio of eugenol, cuminal, and thymol to be 2:2:1. Eugenol (4.00 g), cuminal (4.00 g), and thymol (2.00 g) were weighed according to the mass ratio of 2:2:1 and added to a separate beaker. The mixture was stirred with a glass rod to obtain the composite essential oil (CEO), which was then poured into the beaker containing the wall material solution. The mixture was homogenized at 800 rpm for 6 min using a homogenizer (Model 3000, DREMEL, Mexico City, Mexico) to obtain a homogeneous and stable emulsion. The microencapsulated emulsion was then spray-dried using a spray dryer (SD-BASIC, Labplant, Hunmanby, NY, UK). The spray dryer was set with an inlet air temperature of 180 °C and an outlet air temperature of 80 °C. Once the device reached the set temperature and stabilized, the peristaltic pump was activated, with the flow rate set to 15 mL/min. The emulsion was spray-dried, and the resulting composite EO microcapsule powder was collected and stored in a sealed bag under refrigeration for subsequent experiments. After measurement, in the CEO mps, the content of eugenol was 83.59 μL/g, the content of cuminal was 83.59 μL/g, and the content of thymol was 71.65 μL/g. The amounts of the required experimental materials can be proportionally increased according to need during the experimental process.

### 2.4. Determination of AITC & β-CD Inclusion Characterization

#### 2.4.1. Determination of Nuclear Magnetic Resonance (NMR) Hydrogen Spectra of Inclusions

Five milligrams of β-CD or AITC & β-CD inclusion complexes were dissolved in 0.5 mL of deuterated water (D_2_O). The samples were then analyzed using a 1H NMR spectrometer (NW30VFE, Bruker, Karlsruhe, Germany) to determine the chemical shifts of the H-3 and H-5 peaks in β-CD. The ratio of the area of the H-3 peak to the area of the AITC peak was also calculated. Chemical shifts were referenced to the residual solvent peaks, and all values were reported in parts per million (ppm).

#### 2.4.2. Determination of Inclusion Infrared Spectra (FTIR)

The molecular structures and chemical compositions of the β-CD and AITC & β-CD inclusion complexes were analyzed using an infrared spectrometer (Carry 630, Agilent Technologies Inc., Santa Clara, CA, USA). It was determined by using a Fourier Transform Infrared Spectrometer in the transmission mode. The analytical parameters were set with a resolution of 16 cm^−1^, 64 scans, and a scanning range from 4000 cm^−1^ to 500 cm^−1^. The AITC, β-CD, and AITC & β-CD samples were separately placed in the optical path and scanned for their infrared spectra.

#### 2.4.3. Determination of Encapsulation Constant

The encapsulation constant serves to quantitatively characterize the binding strength between the host and the guest during the formation of inclusion complexes. It mirrors the degree of difficulty in the formation of inclusion complexes and is customarily denoted by “K”. Its calculation method typically involves leveraging the stoichiometric relationships of the equilibrium reaction, in conjunction with thermodynamic data such as enthalpy change and entropy change, for the computation. The determination of the inclusion constant was carried out at five different temperatures: 20 °C, 25 °C, 30 °C, 35 °C, and 40 °C. In cases where the AITC content is excessive, the encapsulation process between β-CD and AITC in solution will reach equilibrium, which can be described by the following equation:

$$\begin{array}{ccccc} \underline{\mathrm{CD}} & + & \underline{\mathrm{Guest}} & = & \underline{\mathrm{CD}•\mathrm{Guest}}\\ ⇓ & & ⇓ & & ⇓\\ a-x & & b-x & & x \end{array}$$

The inclusion constant, i.e., the equilibrium constant, is given by

(1) $$K=\frac{x}{\left(a-x)(b-x\right)}$$

In the formula, “*a*” represents the total concentration of β-CD, “*b*” represents the total concentration of allyl isothiocyanate (AITC), and “*x*” represents the concentration of the AITC & β-CD inclusion complex. If the initial concentration of AITC is denoted as “*S₀*”, then when AITC is encapsulated by β-CD and its solubility increases, the total concentration of AITC in solution is given by “*b = S₀ + x*”. This value is substituted into Equation (1), which leads to the equation

(2) $$K=\frac{b-S_{0}}{\left[a-(b-S_{0})\right]S_{0}}$$

By further manipulation, Equation (2) can be simplified to obtain the equation

(3) $$\;b=\frac{KS_{0}}{1+KS_{0}}a+S_{0}$$

Based on the relationship in Equation (3), a compatibility curve is constructed by plotting the concentration of AITC against the concentration of β-CD. If the compatibility curve forms a straight line, it suggests a 1:1 encapsulation ratio between AITC and β-CD. The encapsulation constant can then be determined from the slope of this line, as represented in the equation

(4) $$K=\frac{slope}{S_{0}(1-slope)}$$

#### 2.4.4. Determination of Thermodynamic Parameters of the Encapsulation Process

Using the van’t Hoff isothermal equation ∆G=−RTlnK and the Gibbs free energy equation ∆G=∆H−T∆S, a functional relationship among the Gibbs free energy change (Δ*G*), enthalpy change (Δ*H*), and entropy change (Δ*S*) can be derived:

(5) $$lnK=\frac{△H}{RT}+\frac{△S}{R}$$

Here, “*R*” represents the gas constant, with a standard value of approximately 8.314 J/(mol∙K), and “*T*” refers to the absolute temperature.

From the equation, it is evident that there is a linear relationship between 1*/T* and *lnK*. By performing linear regression on the equilibrium constants and temperatures of the AITC and β-CD inclusion process, the thermodynamic parameters Δ*H*, Δ*S*, and Δ*G* of the inclusion process can be calculated from the slopes and intercepts of the resulting straight lines. Based on this data, the thermodynamic behavior of the process can be derived.

### 2.5. Characterization of CEO Mps

#### 2.5.1. Determination of Infrared Spectra (FTIR) of Mps

The molecular structures and chemical compositions of the compounds were analyzed using an infrared spectrometer (Carry 630, Agilent, Agilent Technologies Inc., Santa Clara, CA, USA). It was determined by using a Fourier Transform Infrared Spectrometer in the transmission mode. The analytical parameters were set to a resolution of 16 cm^−1^, 64 scans, and a scanning range from 4000 cm^−1^ to 500 cm^−1^. CEO mps, β-CD, gum arabic (GA), eugenol, thymol, cuminal, and AITC compounds were placed separately in the optical path and scanned.

#### 2.5.2. Characterization of Mps by Scanning Electron Microscopy (SEM)

The morphology of the microcapsules was observed using a high-resolution scanning electron microscope with field emission (COXEM EM-30PLUS, COXEM Corporation, Daejeon, Republic of Korea). The microcapsules were deposited on a silicon plate in the wet state and subsequently freeze-dried. The silicon plate containing the microcapsules and other materials was attached to a short brass tube using double-sided tape. A thin layer of gold was sprayed onto the samples, which were then examined using the scanning electron microscope.

#### 2.5.3. Determination of Microcapsule Size Distribution

The particle size distribution of the microcapsules was measured using a laser particle size analyzer (BT-9300H, Shandong Nexter Analytical Instruments Co., Ltd., Jinan, Shandong, China). The microcapsules were thoroughly mixed, and a portion was randomly selected and added to a beaker containing a specific concentration of petroleum ether. The beaker was sonicated for 1 min to ensure proper dispersion. The resulting solution was then transferred to a specialized cuvette for laser particle size scanning.

### 2.6. Gas Chromatography–Mass Spectrometry (GC/MS) Analysis

(1) Instrument Configuration

The substances within CEO mps, as well as their release rates, were analyzed using a GC/MS system [16]. The samples were dissolved in hexane for analysis, employing an Agilent GC/MS instrument, which included a G6501-CTC autosampler, an Agilent 7890 gas chromatograph, an Agilent 5975C mass spectrometer detector (equipped with an electron bombardment ionization source), and an Agilent DB-5MS capillary column (30 m × 0.250 mm).

(2) Chromatographic Conditions

The column temperature was initially set at 40 °C for 8 min, then ramped to 210 °C at a rate of 25 °C per min, and maintained at 210 °C for an additional 5 min. The column chamber was held at 40 °C, with helium used as the carrier gas at a flow rate of 1 mL/min. The split ratio was set at 30:1. The inlet temperature was maintained at 200 °C, while the detector temperature was set to 280 °C. A solvent delay of 4 min was applied for compound identification.

(3) Mass Spectrometry Conditions

The analysis was performed using an Electron Ionization (EI) ion source. The ion source temperature was set to 270 °C, and the interface temperature was also maintained at 270 °C. The ionization voltage was 70 eV, with an emission current of 6 μA and an electron multiplication voltage of 900 V.

**Figure 1:**
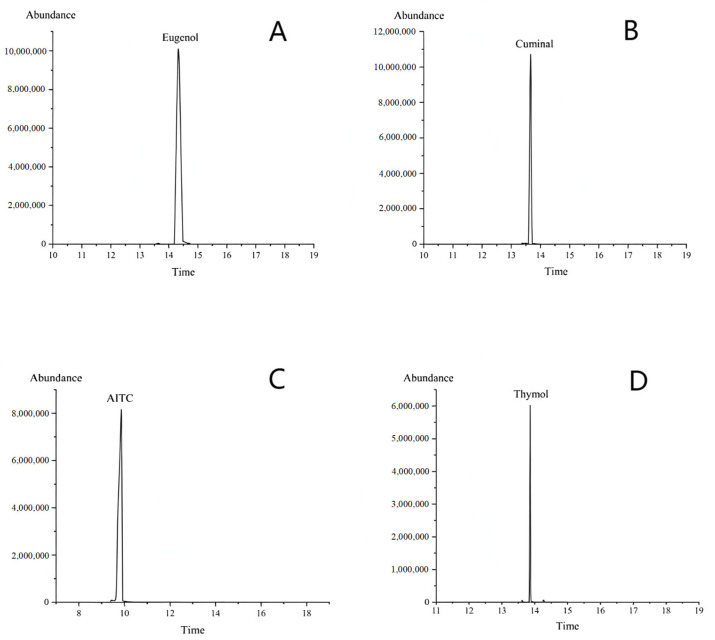
GC/MS total ion chromatogram of standard. (**A**) Eugenol. (**B**) Cuminal. (**C**) AITC. (**D**) Thymol.

The retention times of the different substances were observed to vary: the retention time of the eugenol standard was 14.34 min (Figure 1A), that of the cuminal standard was 13.65 min (Figure 1B), the retention time of the AITC standard was 9.72 min (Figure 1C), and the retention time of the thymol standard was 13.82 min (Figure 1D).

(4) Determination of Standard Curves

Equal volumes (200, 400, 600, 800, 1000 µL) of AITC, eugenol, thymol, and cuminal were accurately transferred into five separate 10 mL volumetric flasks using a pipette. Each flask was filled to volume with n-hexane to prepare standard solutions of EO with concentrations of 20.00, 40.00, 60.00, 80.00, and 100.00 µL/mL. Two milliliters of each standard solution were transferred to capped sample bottles. Each solution was analyzed by GC/MS, and standard curves were constructed.

AITC standard curve: y = 52,856x + 6.0 × 10^6^, R^2^ = 0.9993

Eugenol standard curve: y = 1154.6x + 1.0 × 10^7^, R^2^ = 0.9994

Thymol standard curve: y = 662.23x + 1.7 × 10^5^, R^2^ = 0.9991

Cuminal Standard curve: y = 2,188,100x + 1.2 × 10^7^, R^2^ = 0.999

### 2.7. Application of CEO Mps in Chilled Pork Preservation

#### 2.7.1. Experimental Design for Meat Sample Preparation and Preservation

All knives, cutting boards, and ziplock bags were sterilized with 75% alcohol and exposed to ultraviolet (UV) light for 20 min. The CEO mps, AITC mps, and blank mps were also exposed to UV light for 30 min prior to treatment. Chilled pork was then cut into approximately 50 g cubes and randomly divided into four treatment groups. Samples were collected from each group every 3 days, with three pieces of chilled pork taken from each group on each sampling day [16]. The mps were packed into small pockets (6 cm length, 3 cm width, non-woven fabric). The treatments were as follows: (1) blank: chilled pork; (2) chilled pork + blank microencapsulation package; (3) chilled pork + 0.6% (*w*/*w*) AITC microencapsulation package; and (4) chilled pork + 0.6% (*w*/*w*) CEO microencapsulation package [17]. The sealing line of the slow-release packets was compressed with a sealing strip from a polyethylene self-sealing bag, ensuring the packet hung from the top of the bag to avoid direct contact with the chilled pork. Each sample was packaged individually. The four treatment groups were stored at 0–4 °C and sampled at 0, 3, 6, 9, and 12 days. Statistical analysis of significance was based on comparisons between groups at each sampling time.

#### 2.7.2. pH Measurement

To prepare the samples, 10 g of chilled pork was mixed with 90 mL of distilled water and homogenized for 20 min. The mixture was then centrifuged at 6000 rpm for 10 min, and the supernatant was collected. The pH of the supernatant was measured three times using a pH meter (FE20, Mettler-Toledo International Inc., Greifensee, ZH, Switzerland).

#### 2.7.3. Color Determination

The color of the samples was measured using a colorimeter (LS-175, Shenzhen Linshang Technology Co., Ltd., Shenzhen, China) with a 20 mm aperture. Before each measurement, a white tile was used for calibration. To ensure accurate readings, the samples were rotated 60° clockwise to eliminate any aperture between the meat sample and the measuring head. Five measurements were taken for each sample. The color measurements were expressed using the CIE LAB (L*, a*, b*) color scale, where L* represents brightness, a* indicates redness, and b* indicates yellowness.

#### 2.7.4. Thiobarbituric Acid Reactive Substances (TBARSs)

Lipid oxidation was assessed using the TBARS method as described by Fan et al. [18]. Ten grams of ground meat were homogenized with 50 mL of 7.5% trichloroacetic acid (TCA) containing 0.1% ethylenediaminetetraacetic acid. The mixture was shaken for 30 min and then filtered. Five milliliters of the filtrate were mixed with an equal volume of 2-thiobarbituric acid (TBA) (0.02 mol/L). The resulting mixture was heated at 100 °C for 40 min, cooled for 1 h to room temperature, and then centrifuged at 6000 rpm for 5 min. Five milliliters of chloroform were added to the supernatant, which was then shaken well. After dispensing, the absorbance of the supernatant was measured at A532 nm and A600 nm. Blank controls consisted of 5 mL TBA and 5 mL TCA. The TBARS content was calculated using the following formula and expressed as mg malondialdehyde (MDA) per kg of meat:

(6) $$TBARS(mg/kg)=\frac{(A_{532}-A_{600})\times V\times M}{155\times 10}$$

where *A*_532_ is the absorbance value at 532 nm wavelength, *A*_600_ is the absorbance value at 600 nm wavelength, “*V*” is the volume of the supernatant (mL), and “*M*” is the molar mass of malondialdehyde (72.6 g/mol).

#### 2.7.5. Total Volatile Base Nitrogen (TVB-N)

Initially, 10 g of minced meat was homogenized in 75 mL of deionized water and left to stand for 30 min prior to filtration. The resultant filtrate was combined with 1 g of magnesium oxide (used as a catalyst) in a digestion tube and immediately distilled using an automatic Kjeldahl nitrogen analyzer (K1100, Haineng Future Technology Group Co., Ltd, Shandong, Jinan, China). The distillate was subsequently titrated with 0.1 M HCl. The volatile saline nitrogen content (X) of the samples was calculated using the following formula:

(7) $$H=\frac{(V1-V2)\times c\times 14}{m}\times 100$$

where: “*H*” represents the content in mg/100 g or mg/100 mL; “*V*1 ”denotes the volume of hydrochloric acid or sulfuric acid standard titration solution consumed by the test solution (mL); “*V*2” denotes the volume of hydrochloric acid or sulfuric acid standard titration solution consumed by the reagent blank (mL); “*c*” signifies the concentration of hydrochloric acid or sulfuric acid standard titration solution (mol/L); “*m*” represents the mass of the meat (g).

#### 2.7.6. Measurement Methods of Water Loss Rate

The initial mass of each piece of chilled pork and the associated ziplock bag was measured. Each day, the chilled pork was removed from the ziplock bag and left to dry in a ventilated area for 30 s. Subsequently, the total mass of the ziplock bag and the chilled pork sample was recorded. The water loss rate was calculated using the following formula:

(8) $$Loss\;of\;water\;(\%)=\frac{M+m-W}{M+m}\times 100\%$$

where: “*M*” represents the initial mass of the chilled pork (g); “*m*” represents the initial mass of the ziplock bag (g); “*W*” denotes the total mass of the ziplock bag and the chilled pork sample (g).

#### 2.7.7. Measurement Methods of Total Colony Count

The experimental procedure followed the method outlined in Ref. [19]. For the homogenization of the pork samples, 10 g of chilled pork was combined with 90 mL of sterile water and homogenized for 3 min. A 10-fold serial dilution of the homogenate was then prepared. A 100 μL aliquot of the diluted homogenate was spread onto an agar plate and incubated at 37 °C for 48 h for total bacterial count (TBC) determination.

### 2.8. Statistical Analysis

All experiments and measurements were performed in triplicate, and the results are expressed as mean ± standard deviation (SD). Data were processed and visualized using Origin 2017 and Microsoft Excel spreadsheet software. Statistical analyses were conducted using SPSS version 26.0 (SPSS Inc., Chicago, IL, USA). Significant differences were determined using the least significant difference (LSD) test at the 5% significance level (*p* < 0.05).

## 3. Results and Discussion

### 3.1. Characterization of AITC and β-CD Inclusion Complexes

#### 3.1.1. NMR Hydrogen Spectroscopy

**Figure 2:**
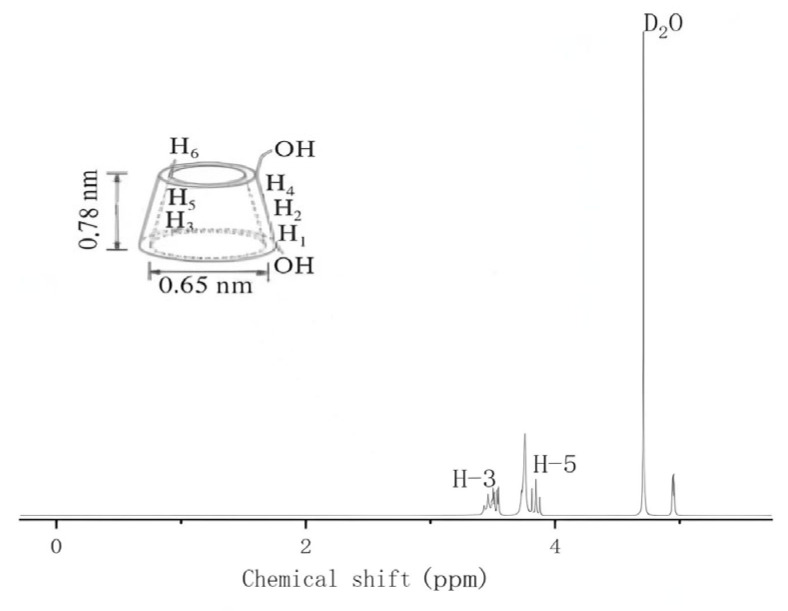
β-CD one-dimensional NMR spectroscopy.

**Figure 3:**
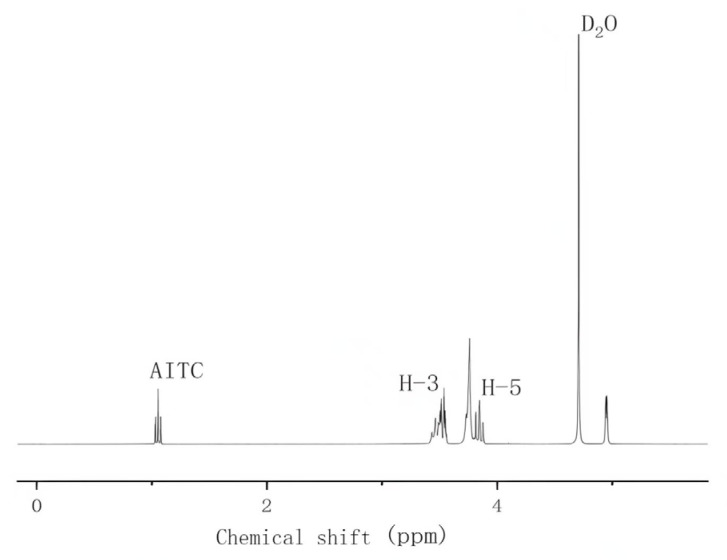
β-CD clathrate one-dimensional NMR spectroscopy.

In order to study the possible inclusion mode of AITC and β-CD, we compared the one-dimensional nuclear magnetic resonance spectra of β-CD (as shown in Figure 2) and the inclusion complex (as shown in Figure 3). Compared with DMSO, deuterium oxide (D_2_O), which is more commonly used and has no interfering effects, was selected as the solvent to dissolve the pure β-CD and the inclusion complex.

Table 1 illustrates the variations in the chemical shift values of the main groups of β-CD before and after inclusion, as observed in the one-dimensional NMR hydrogen spectra. The data indicate that the chemical shifts of H-3 and H-5, which are located within the hydrophobic cavity of β-CD, were shifted to lower fields, whereas H-1 and H-4, situated outside the cavity, remained unaffected. This phenomenon can be attributed to the partial entry of AITC molecules into the hydrophobic cavity of β-CD, leading to changes caused by the shielding effect of the chemical environment, thereby confirming the formation of inclusion complexes between AITC and β-CD. Additionally, the change in the H-3 chemical shift was more pronounced than that of H-5, further corroborating that AITC enters the cavity through the hydrophobic end (i.e., the wide-mouth end) of β-CD.

By analyzing the area ratio of the characteristic peaks, the actual inclusion molar ratio was determined. The integral peak of AITC was automatically calculated by setting the area of the H-3 peak to 1. The ratio of the area of the characteristic peak of AITC to the area of the primary characteristic peak of β-CD was 1:1.03, indicating that β-CD encapsulates AITC in a 1:1 molar ratio.

#### 3.1.2. FTIR Spectroscopy of β-CD Inclusion Complex

**Figure 4:**
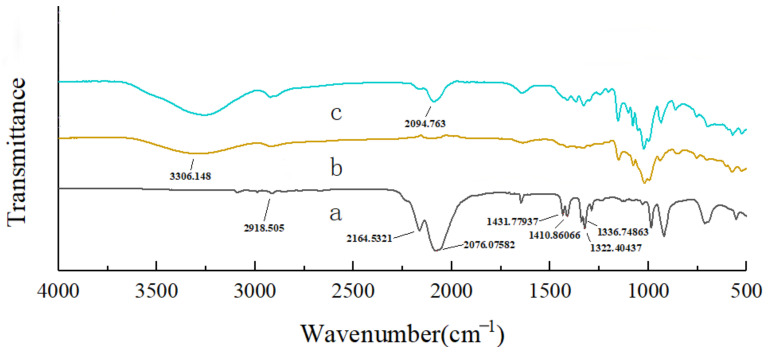
FTIR spectral analysis. a: AITC, b: β-CD, c: AITC & β-CD inclusion complexes.

The FTIR spectrum of the AITC, β-CD and β-CD inclusion complex displayed a characteristic absorbance at 2094.763 cm^−1^, corresponding to the -N=C=S bond in the AITC molecule (Figure 4) This signal was weaker than in AITC alone, indicating that AITC was successfully encapsulated by β-CD.

#### 3.1.3. Determination of Inclusion Constants

**Figure 5:**
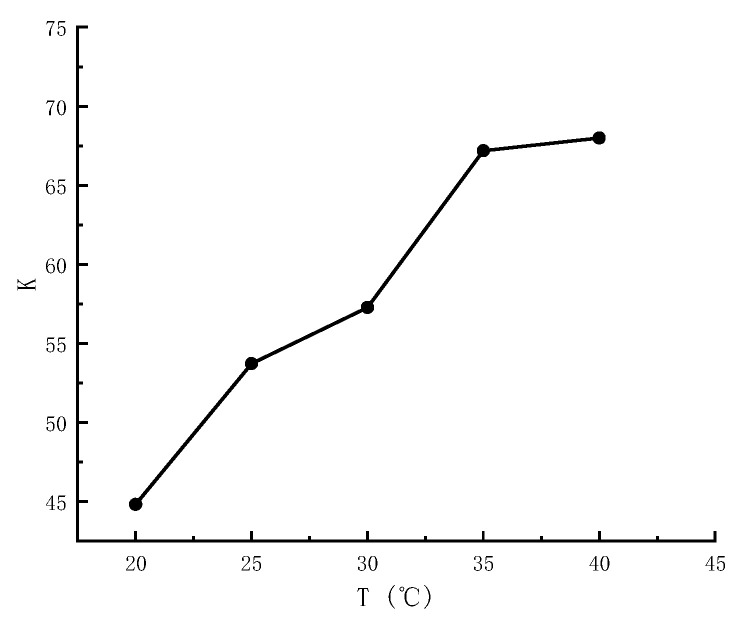
Cladding constants of β-CD and AITC in phase solubility experiments at five temperatures.

As shown in Figure 5, the inclusion constant, or equilibrium constant, increased with rising temperature, indicating that the maximum K value and the strongest inclusion occurred at 40 °C. The observed increase in the inclusion values of AITC and β-CD with increasing temperature may be attributed to strong intermolecular forces, particularly hydrogen bonding, at elevated temperatures, which enhanced the encapsulation effect.

#### 3.1.4. Calculation of Thermodynamic Parameters

**Figure 6:**
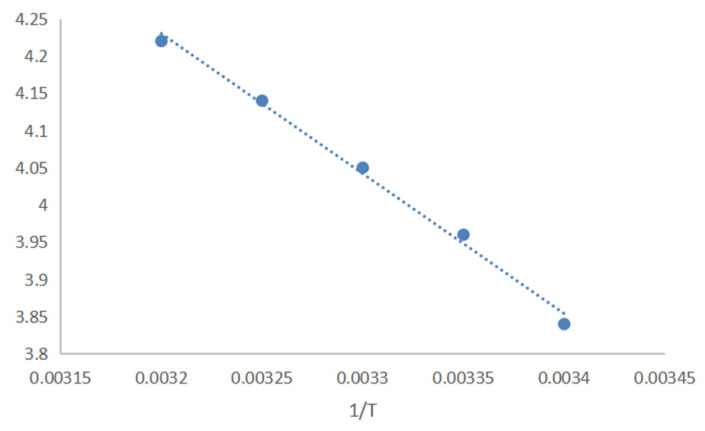
1/T of AITC and β-CD inclusions corresponding to lnK at different temperatures.

**Figure 7:**
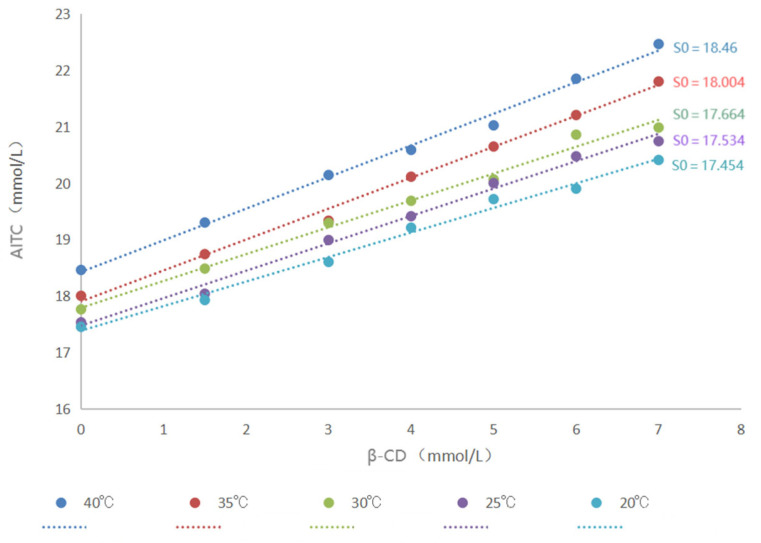
Phase solubility curves of AITC & β-CD inclusion complexes.

Figure 6 presents the results of linear fitting and regression of the natural logarithm of the equilibrium constant for the β-CD encapsulated AITC process against the reciprocal of temperature, with the regression equation y = −1880x + 4.33 and an R^2^ value of 0.9941 at different temperatures. The slope of the equation was −1880, and the intercept was 10.246. The enthalpy change ΔH was calculated as −16.17 kJ/mol, the entropy change ΔS as 87.10 J/mol, and the Gibbs free energy ΔG as −9.26, −9.87, −10.20, −10.77 and −10.98 kJ/mol at 20, 25, 30, 35, and 40 °C, respectively (Table 2, Figure 7) Negative Gibbs free energy ΔG indicates that the inclusion process is spontaneous; negative enthalpy change ΔH signifies that the process is exothermic; and positive entropy change ΔS indicates that the process occurs without an increase in system order.

### 3.2. Morphological Properties of CEO Mps

#### 3.2.1. FTIR Spectroscopy

**Figure 8:**
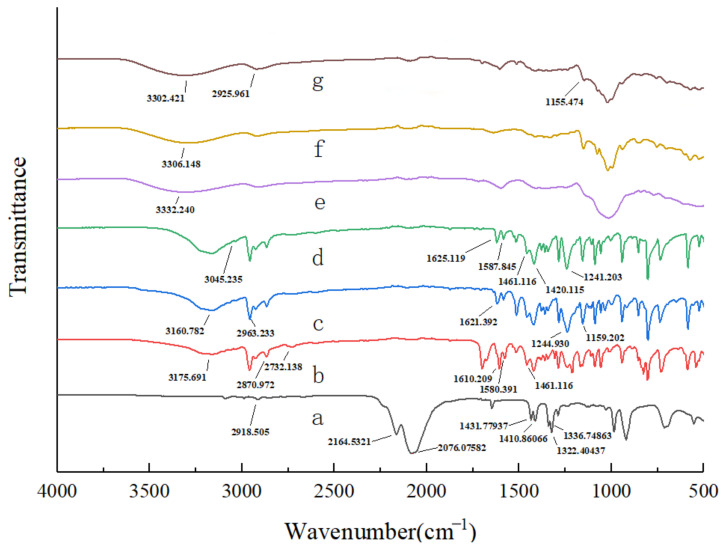
FTIR spectral analysis. a: AITC. b: Thymol. c: Eugenol. d: Cuminal. e: Gum arabic. f: β-CD. g: CEO mps.

The structural differences among CEO mps, β-CD, GA, cuminal, eugenol, thymol, and AITC are presented in Figure 8. In the FTIR spectra of AITC, wavenumber at 2167.491 cm^−1^ and 2083.294 cm^−1^ were assigned to the -N=C=S stretching vibration. The wavenumber at 1286.039 cm^−1^ was associated with C-N bonding. CH deformation vibrations (3δ for CH) produced wavenumber at 1436.549, 1416.431, and 1327.019 cm^−1^, while the C-H deformation vibration due to =CH was observed at 1347.137 cm^−1^. In the spectrum of cuminal, hydrocarbon stretching vibrations on the benzene ring were observed at 3175.691 cm^−1^, with wavenumber at 2870.972 cm^−1^ and 2732.138 cm^−1^ indicating the C-H stretching vibration associated with the carbonyl carbon. The carbonyl carbon-oxygen stretching vibration occurred at 1700.639 cm^−1^, and the carbonyl carbon stretching vibration was observed at 1610.209 cm^−1^. Wavenumber at 1610.209 cm^−1^, 1580.391 cm^−1^, and 1461.116 cm^−1^ corresponded to the backbone vibration of the benzene ring.

The FTIR spectra of eugenol at 3160.782 cm^−1^, 2963.233 cm^−1^, 1621.392 cm^−1^, and 1244.930−1159.202 cm^−1^ displayed wavenumber corresponding to -OH, -C-H, -C=C-, and -C-O-C- groups, respectively, consistent with previous reports [19]. In the spectrum of thymol, the wavenumber at 3045.235 cm^−1^ was related to the stretching vibration of C-H in the benzene ring. Wavenumber at 1625.119 cm^−1^, 1587.845 cm^−1^, 1519.906 cm^−1^, and 1461.116 cm^−1^, of varying intensities, corresponded to the characteristic of muscovite phenol. Wavenumber at 1420.115 cm^−1^ and 1241.203 cm^−1^ were associated with the bending vibrations of -OH and C-O, respectively.

In the CEO mps, the wavenumber at 2918.505 cm^−1^ disappeared, indicating that the -CH_2_- group was encapsulated within the host cavity. The FTIR spectra of the mps displayed wavenumber at 2925.961 cm^−1^ and 1155.474 cm^−1^, suggesting the successful encapsulation of eugenol and thymol into the CEO mps, respectively. The -OH-related wavenumber at 3302.421 cm^−1^ in the CEO mps was shifted to lower wavelengths compared to β-CD (3306.148 cm^−1^) and GA (3332.240 cm^−1^), due to hydrogen bonding between the phenols and the wall material [20]. Additionally, the characteristic wavenumber of C-N bonds disappeared, as their stretching vibrations were restricted following the formation of the CEO mps. Infrared spectroscopy thus confirmed that CEO mps was successfully encapsulated within the composite wall material.

#### 3.2.2. SEM

**Figure 9:**
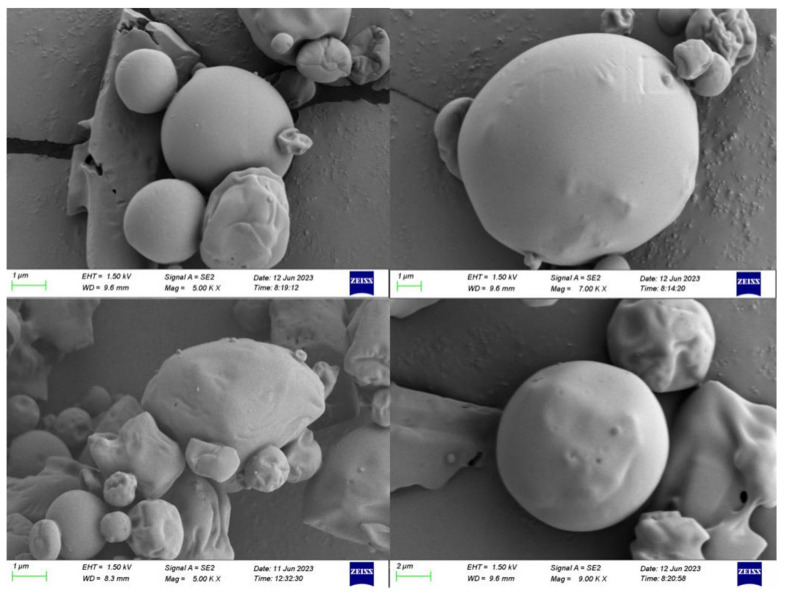
SEM image of complex plant essential oil microcapsules. (In the four images from left to right and top to bottom, the scale bars are 1 μm, 1 μm, 1 μm, and 2 μm respectively).

As shown in Figure 9, the spherical shape of the microcapsule particles, produced by spray drying at different magnifications with slight concavity, was a common feature of spray-dried materials. This concavity may result from the high inlet temperature of the spray dryer and material shrinkage due to the rapid evaporation of the wall material during the drying process [21]. Furthermore, no cracks or visible pores were observed on the surface of the CEO mps, a characteristic that aids in the preservation of volatile compounds within the CEO mps, reduces the permeability to oxidizing gases, and improves encapsulation efficiency.

#### 3.2.3. Particle Size and Physical Properties

**Figure 10:**
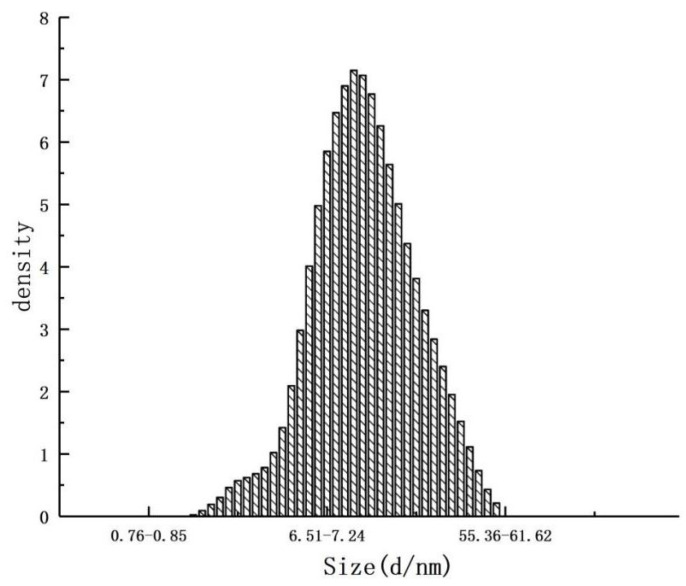
Particle size distribution of CEO mps.

The uniformity and flowability of microencapsulated particles can significantly influence their effectiveness in food applications. The properties of these microencapsulated particles (CEO mps) are impacted by the size of the powder particles. Larger particles, when added, can affect the texture of the food product [22]. Figure 10 shows that the minimum particle size of CEO mps ranged from 1.31 to 1.45 μm, while the maximum particle size ranged from 49.74 to 55.36 μm. The largest proportion of particles fell within the size range of 8.97 to 9.98 μm, comprising 7.15%, followed by particles in the 9.98 to 11.11 μm range at 7.05%. Similar droplet sizes were observed in juniper berry essential oil (JBEO) prepared using GA maltodextrin (MD) mps [19]. These results suggest that CEO mps are suitable for the preservation of essential oils (EOs).

**Figure 11:**
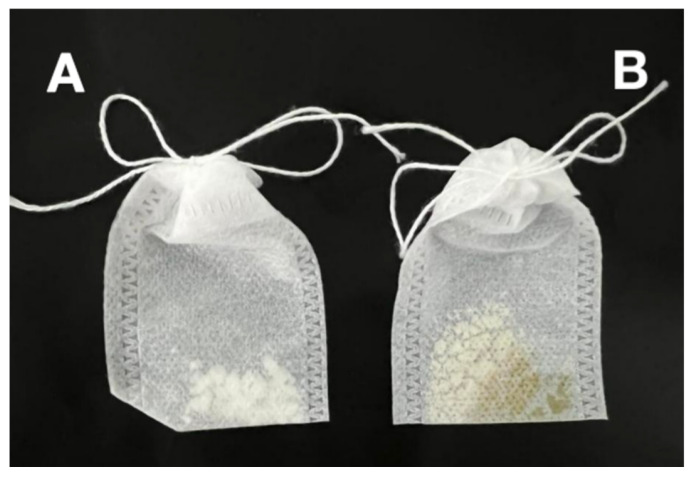
Comparison of mps appearance CEO mps (**A**) AITC&β-CD inclusion complexes (**B**).

Figure 11 illustrates the CEO mps stored in a 6-day slow-release bag. The non-woven slow-release bag is a carrier with a porous structure. We conducted a comparative experiment by placing CEO mps in an open container and in a non-woven slow-release bag. The results showed that there was almost no significant difference in the effects, and the non-woven slow-release bag proved to be a more favorable application method. It is evident that the CEO mps exhibited a superior appearance compared to AITC inclusion complexes. The CEO mps overcame issues such as caking, yellowing, and stickiness observed in AITC mps. This improvement can primarily be attributed to the encapsulation of AITC within the β-CD cavity, which reduced the ability of β-CD to bind to water molecules.

### 3.3. Slow-Release Properties of CEO Mps

**Figure 12:**
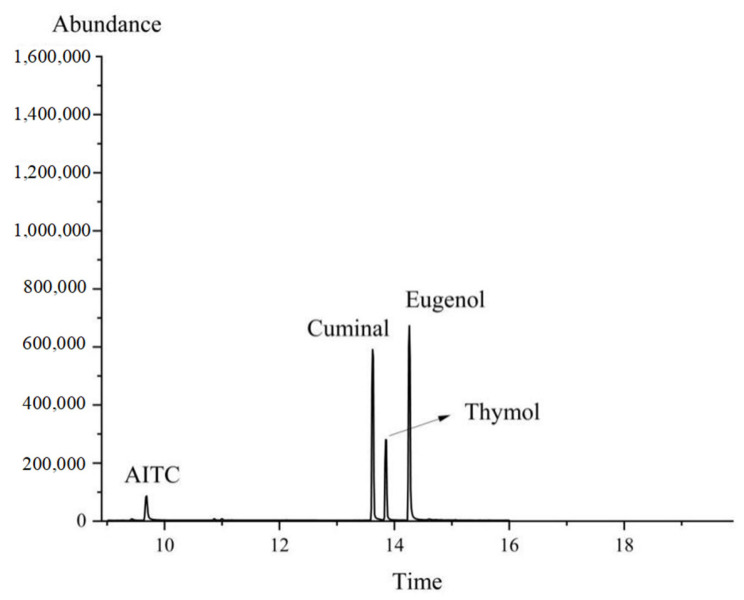
GC/MS total ion chromatogram of CEO mps.

**Figure 13:**
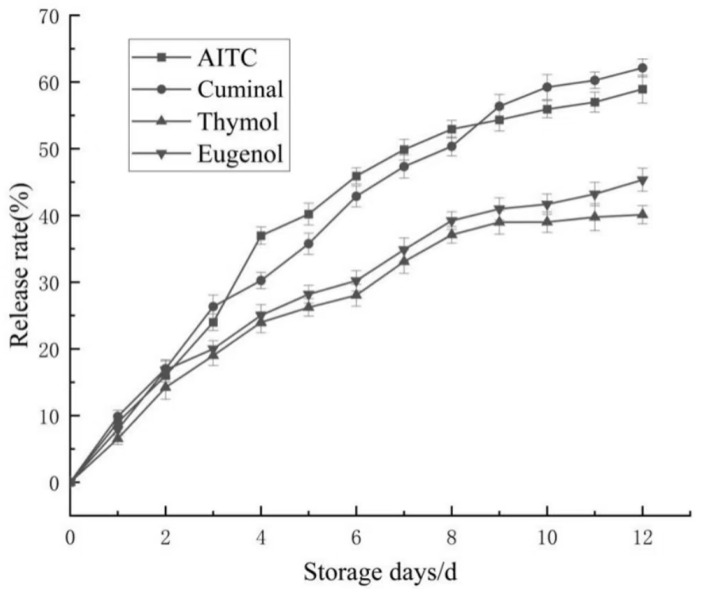
Release rate of CEO mps.

Figure 12 presents the four compounds detected from the CEO mps using GC-MS. Figure 13 shows the release rates of AITC, eugenol, cuminal, and thymol from the CEO mps. The release rates during the first 8 days were 52.51%, 49.75%, 37.09%, and 39.23%, respectively, and equilibrium was reached by the 9th day. Additionally, the cumulative release of these compounds by the 12th day was 61.82%, 57.96%, 44.34%, and 38.65%, respectively. These findings indicate that the CEO mps exhibited optimal slow-release characteristics.

### 3.4. Application of CEO Mps in Chilled Pork

#### 3.4.1. pH

**Figure 14:**
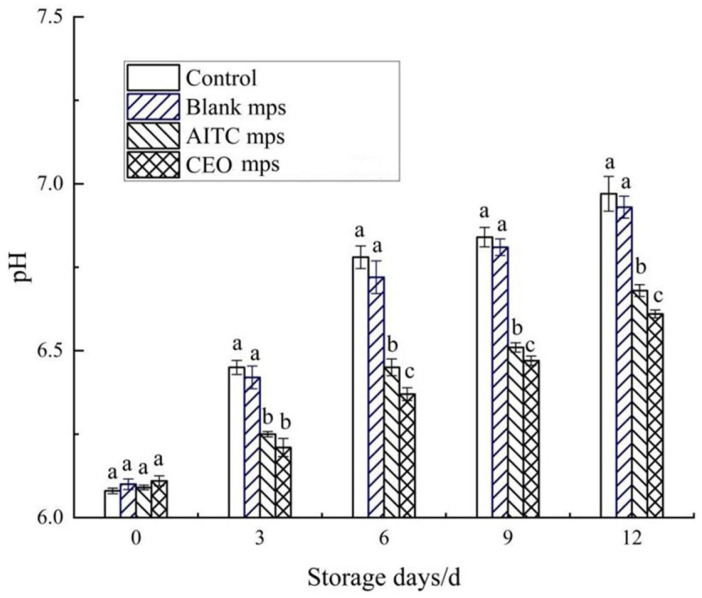
Changes in pH value of chilled pork during storage. (The same letters indicate non-significant differences, while different letters indicate significant differences).

A strong correlation has been established between the pH of meat and microbial activity. Microorganisms and enzymes break down proteins in meat into basic ammonia and amines during storage, which accumulate over time, leading to an increase in pH in pork [16,23,24]. The initial pH of chilled pork ranged from 5.8 to 6.2, while spoiled pork exhibited a pH greater than 6.7. As shown in Figure 14, the pH of the control groups was slightly higher than that of the treated groups on the 3rd day of storage. No significant differences (*p* > 0.05) in pH were observed between the different treatment groups on the 3rd day. After 3 days of storage, the pH of all samples gradually increased. The blank and blank microencapsulated groups showed signs of deterioration after the 6th day (6.78 ± 0.034 and 6.72 ± 0.049, respectively), whereas the pH of the AITC and CEO microencapsulation groups did not exceed 6.7 until the 12th day. These findings suggest that the use of CEO mps effectively delayed the increase in pH of pork during cold storage. While AITC mps also delayed the increase in pH, their effectiveness was slightly lower compared to CEO mps.

#### 3.4.2. Color Measurement

**Figure 15:**
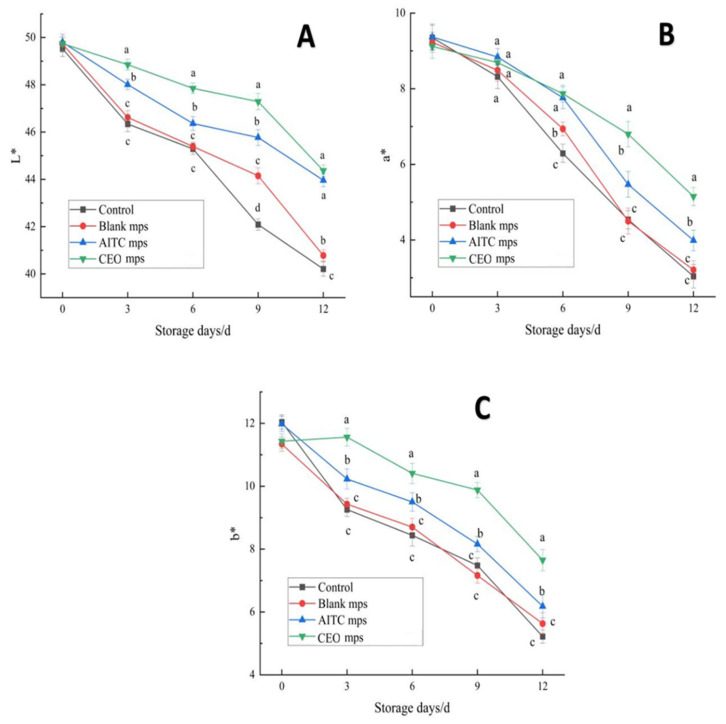
Color change of chilled pork during storage. L* value change (**A**), a* value change (**B**), b* value change (**C**). (The same letters indicate non-significant differences, while different letters indicate significant differences).

The color of chilled pork plays a crucial role in determining consumer preference and purchasing decisions. Figure 15A shows the change in the L* value, which represents the gloss of the pork, during the storage period. As observed, the L* values of all treatment groups decreased over time, with the blank control and blank microencapsulated control groups exhibiting the fastest declines, reaching 40.21 ± 0.31 and 40.78 ± 0.21 on the 12th day, respectively. In contrast, the AITC and CEO mps treatment groups exhibited slower decreases, with L* values of 43.96 ± 0.27 and 44.37 ± 0.14, respectively. Although the differences between the treatment groups were not statistically significant (*p* > 0.05), both treatment groups showed significant differences (*p* < 0.05) compared to the blank control group, indicating that the microencapsulated systems effectively protected the color of chilled pork.

Figure 15B shows the a* value, which represents the red coloration of pork during storage. The a* value of the blank control group decreased to 3.04 ± 0.31 on the 12th day, followed by the blank microencapsulated control group (3.21 ± 0.24). There were no significant differences between these two groups. However, on the 12th day, the a* values for the AITC microencapsulated group and the CEO mps group were 3.99 ± 0.27 and 5.15 ± 0.22, respectively, both of which were significantly different from the blank control group.

Figure 15C shows the variation in the b* value during the storage of chilled pork, which indicates the degree of yellowing and discoloration of the meat due to the oxidation of myoglobin to metmyoglobin on the surface of the pork. As observed, except for the CEO mps group, which showed a tendency to increase and then decrease, the b* values of the other treatment groups consistently decreased. On the 12th day, the b* value of the CEO mps group was 7.65 ± 0.34, which was significantly different from the other three groups (*p* < 0.05). Based on these results, it can be concluded that the CEO mps group effectively delayed the color change of chilled pork during the storage period, exhibiting superior performance in preserving the color of the meat.

#### 3.4.3. TBARS

**Figure 16:**
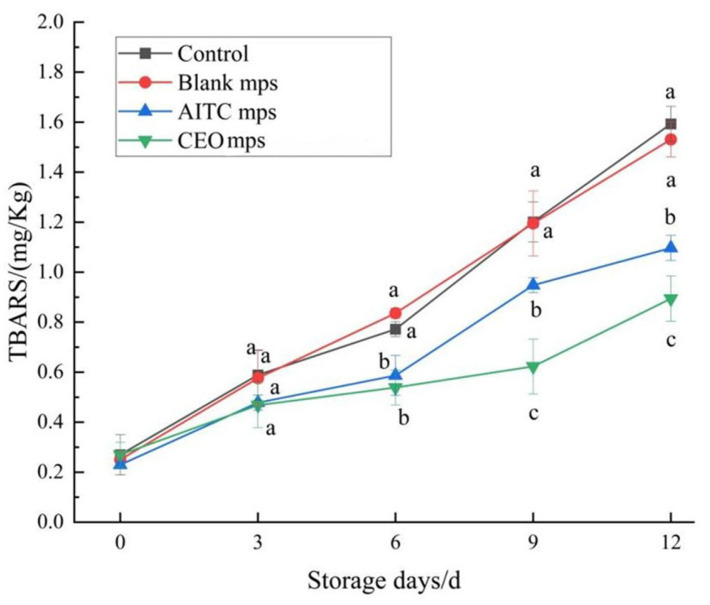
Changes in TBARS values of chilled pork during storage. (The same letters indicate non-significant differences, while different letters indicate significant differences).

Lipid oxidation is a key factor contributing to the deterioration of quality in chilled pork. The thiobarbituric acid reactive substances (TBARSs) assay is the most widely used method for assessing lipid oxidation [25,26]. This method quantifies the degree of fat oxidation by measuring the amount of lipid peroxides and malondialdehyde, a by-product of peroxidation, formed during the oxidation of polyunsaturated fatty acids. As shown in Figure 16, the initial TBARS values for all groups were approximately 0.2 mg MDA/kg. On the 3rd day, the TBARS values for the control and blank microencapsulated control groups increased significantly to 0.58 ± 0.10 mg MDA/kg and 0.57 ± 0.11 mg MDA/kg, respectively. The TBARS value of the AITC microencapsulated treatment group also increased significantly from the 9th day onward, reaching 0.94 ± 0.03 mg MDA/kg. The TBARS value for the CEO microencapsulation-treated group remained significantly lower than those of the other groups, reaching 0.69 ± 0.07 mg MDA/kg by the 12th day (*p* < 0.05). These results suggest that the CEO mps significantly reduced the degree of fat oxidation in meat throughout the storage period. Compared to our previous study [5], the continued use of AITC, which possesses strong bacteriostatic properties, along with the addition of CEO with antioxidant activity, provided both bacteriostatic and antioxidant effects, effectively extending the shelf life of chilled pork.

#### 3.4.4. TVB-N

**Figure 17:**
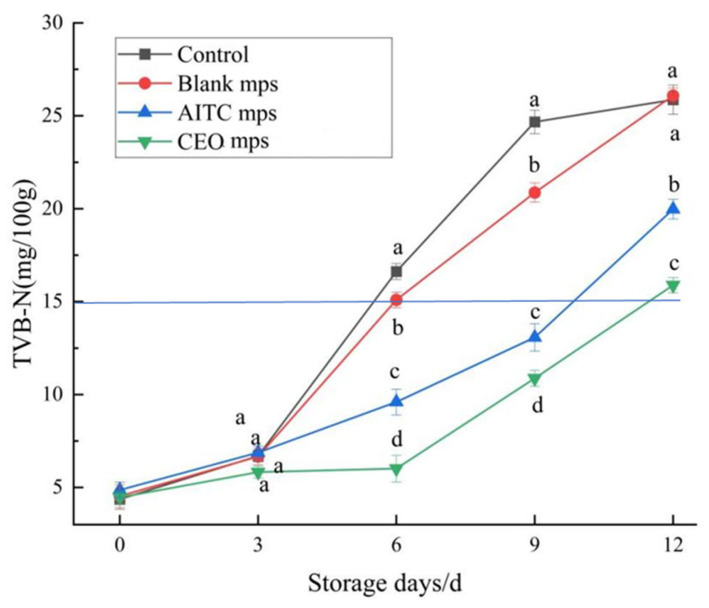
Changes in TVB-N values of chilled pork during storage. (The same letters indicate non-significant differences, while different letters indicate significant differences). (According to GB 2707-2016 National Food Safety Standard for Fresh (Frozen) Livestock and Poultry Products, the national standard limit range of volatile basic nitrogen in pork is ≤15mg/100g).

Total volatile base nitrogen (TVB-N) is an indicator used to assess the freshness of meat and fish products. Higher TVB-N levels (mg/100 g) indicate reduced freshness of these products [27]. According to the Chinese standard GB/T9959.2-2008 for Fresh and Frozen Lean Pork, frozen pork is considered spoiled if its TVB-N exceeds the upper limit of 15 mg per 100 g. Figure 17 shows the TVB-N values of the four treatments. During the first 3 days of storage, the TVB-N values of chilled pork in all treatment groups ranged from 4.37 to 4.87 mg/100 g, with no significant differences between the groups (*p* > 0.05). With the extension of storage time, the release of active substances from the microencapsulated systems effectively inhibited the formation of amines. The TVB-N value of the CEO mps treatment group did not exceed 15 mg/100 g until the 12th day, while the TVB-N value of the control group reached 15 mg/100 g by the 6th day. These results demonstrate that CEO mps effectively delayed the increase in TVB-N value and significantly prolonged the storage life of chilled pork by 6 days during cold storage, with a notable effect [26].

#### 3.4.5. Water Loss Rate

**Figure 18:**
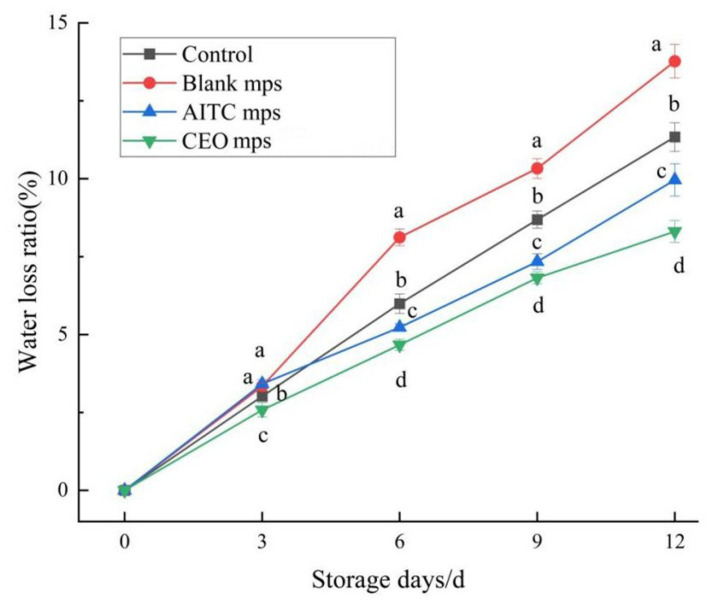
Water loss of chilled pork during storage. (The same letters indicate non-significant differences, while different letters indicate significant differences).

The water loss rate significantly affects the tenderness, color, and overall commercial value of chilled pork. As shown in Figure 18, the water loss rate of the different treatment groups gradually increased with the extension of storage time. On the 12th day, the water loss rates of the AITC microencapsulated and CEO mps treatment groups were significantly different (*p* < 0.05) from those of the blank control group and the blank microencapsulated control group, with values of 9.96 ± 0.32% and 8.31 ± 0.35%, respectively. Moreover, the CEO mps treatment group exhibited a significant advantage over the AITC microencapsulated treatment group, indicating that CEO mps is more suitable for the preservation of chilled pork.

#### 3.4.6. Total Colony Count

**Figure 19:**
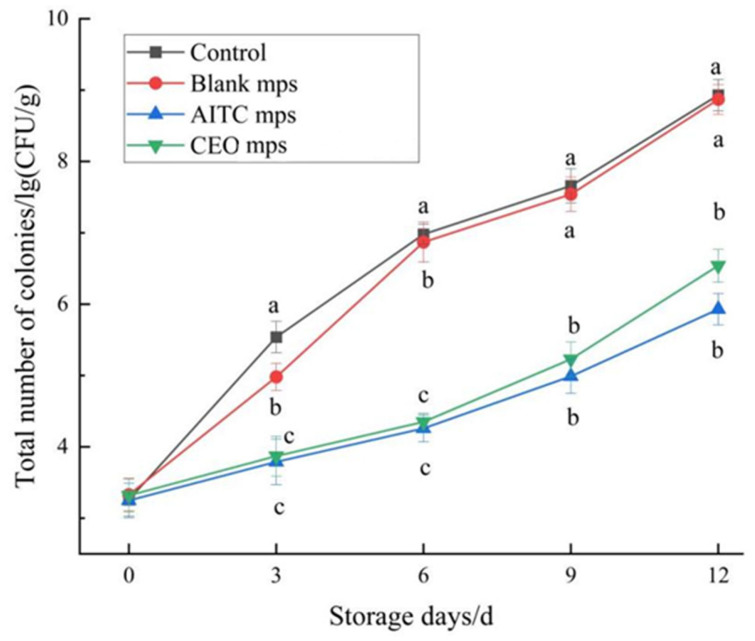
Changes in total colony counts of chilled pork during storage. (The same letters indicate non-significant differences, while different letters indicate significant differences).

Total colony count is a critical indicator of microbial growth in chilled pork during storage [4]. Figure 19 illustrates the increase in total colony count over time. On the 6th day, the total colony counts in the blank control group and the blank microencapsulated control group were 6.98 ± 0.14 log (CFU/g) and 6.87 ± 0.28 log (CFU/g), respectively. In contrast, the total colony counts in the AITC and CEO microencapsulated treatment groups were 4.26 ± 0.19 log (CFU/g) and 4.35 ± 0.12 log (CFU/g), respectively, indicating that the meat quality in the control groups had deteriorated, consistent with the findings of a previous study by Ma et al. [1]. On the 12th day, the total colony counts in the AITC microencapsulated and CEO microencapsulated treatment groups had increased to 5.93 ± 0.22 log (CFU/g) and 6.54 ± 0.23 log (CFU/g), respectively. These values were lower than those in the control groups, demonstrating the effective preservation effect of the microencapsulated treatments. The bacterial inhibition effect of the treatment groups was ranked as follows: blank < blank microencapsulated < CEO microencapsulated < AITC microencapsulated. Although the AITC microencapsulated treatment group exhibited slightly higher inhibition, this was likely due to the higher AITC content in this formulation compared to the CEO microencapsulated treatment group. Nevertheless, the increase in total colony counts in both the AITC and CEO microencapsulated groups was significantly lower than that in the blank microencapsulated group, indicating that both treatments were effective in prolonging the shelf life of chilled pork.

## 4. Conclusions

In this study, a novel microencapsulation system was designed to effectively extend the shelf life of chilled pork while maintaining its quality in terms of pH, color, TBARSs, TVB-N, water loss rate, and total colony counts throughout the storage period. Building on previous research [5], this microcapsule featured a more complex structure and composition. While AITC is known for its excellent bacteriostatic activity, it lacks significant antioxidant properties. Therefore, three plant essential oil (EO) components—eugenol, cuminal, and thymol—were incorporated into the formulation to enhance antioxidant activity and synergistically improve bacterial inhibition with AITC. Through the comparative experiment on the antioxidant effects of single plant essential oils and compound plant essential oils, as well as the proportion optimization experiment, we finally determined the mass ratio of eugenol, cuminal, and thymol to be 2:2:1, with it providing the best combination of antioxidant and bacteriostatic effects.

To ensure the effectiveness of the microencapsulation system in prolonging the shelf life of chilled pork, AITC and the EOs were encapsulated within a β-CD inclusion complex, with the CEO (eugenol–cuminal–thymol) mixture encapsulated using the AITC & β-CD inclusion compound and GA as the wall material to form microcapsules. The resulting microcapsules allowed AITC to remain available for stable release without occupying the internal space, thus facilitating the encapsulation of additional plant EOs. Characterization and evaluation of the microcapsules demonstrated that the size and morphology of the microcapsules were ideal, and the CEO microcapsules prepared with the AITC&β-CD inclusion and GA mixture effectively controlled the release of the core materials. Furthermore, AITC encapsulated in β-CD ensured its stability and sustained release, exerting its bacteriostatic effect over time. The CEO microcapsules effectively extended the shelf life of chilled pork by approximately 12 days and exhibited a strong preservation effect.

The CEO microcapsules developed in this study offer promising potential as a natural preservative for extending the shelf life and improving the quality of refrigerated fresh pork. Additionally, the AITC&β-CD inclusion complex used as the microencapsulation wall material provides a novel approach to microencapsulation design, which could be applied in other fields as well.

## Figures and Tables

**Table 1:** Variation of chemical shift values of major functional groups in one-dimensional NMR spectra of β-CD and β-CD inclusions.

H	Δ (ppm)	
	β-CD	AITC*&*β-CD (Inclusion Complexes)
H_1_	4.94	4.94
H_2_	3.51	3.51
H_3_	3.87	3.82
H_4_	3.45	3.46
H_5_	3.76	3.74
H_6_	3.72	3.73

**Table 2:** Thermodynamic constants of β-CD-coated AITC at five temperatures.

T (°C)	ΔG (kJ/mol)	ΔH (kJ/mol)	ΔS(J/mol)
20	−9.26		
25	−9.87		
30	−10.20	−16.17	87.10
35	−10.77		
40	−10.98		

## Data Availability

The original contributions presented in the study are included in the article, further inquiries can be directed to the corresponding author.
